# Family-based intervention using face-to-face sessions and social media to improve Malay primary school children’s adiposity: a randomized controlled field trial of the Malaysian REDUCE programme

**DOI:** 10.1186/s12937-018-0379-1

**Published:** 2018-08-02

**Authors:** Norliza Ahmad, Zalilah Mohd Shariff, Firdaus Mukhtar, Munn-Sann Lye

**Affiliations:** 1 Department of Community Health, Faculty of Medicine and Health Sciences, Universiti Putra Malaysia, Level 1, Block B (Academic), 43400 UPM, Serdang, Selangor Malaysia; 2 Department of Nutrition, Faculty of Medicine and Health Sciences, Universiti Putra Malaysia, Serdang, Selangor Malaysia; 3 Department of Psychiatry, Faculty of Medicine and Health Sciences, Universiti Putra Malaysia, Serdang, Selangor Malaysia

**Keywords:** Paediatric obesity, Social media, Body mass index, Waist circumference, Body fat percentage, Parents intervention, Primary school children

## Abstract

**Background:**

Social media may be an effective medium by which parents could be trained to promote healthy eating behaviour and physical activity for their children. This trial evaluates the effectiveness of a family-based intervention using social media in combination with face-to-face sessions - the REDUCE (REorganise Diet, Unnecessary sCreen time and Exercise) programme - on adiposity of Malay children.

**Methods:**

Five primary schools in an urban area in Selangor, Malaysia participated in this two-arm randomized controlled field trial. Participants were parents (*n* = 134) and their primary school-going children 8–11 years of age who were either overweight or obese. These parent-child dyads were randomly allocated to intervention and wait-list control groups and were blinded to group assignment. The intervention was a four-week training programme using two face-to-face sessions and two Facebook sessions followed by weekly booster sessions over a three-month period using WhatsApp. The primary outcome was body mass index (BMI) z-score. Height, body weight, waist circumference and percentage of body fat were measured by blinded assessors. Data were collected at baseline (T1), immediately post-training (T2) and at three- (T3) and six-month post training (T4) and were analysed using generalized linear mixed modelling adjusted for covariates to estimate the intervention effects. Subgroup analysis was conducted for overweight and obese children.

**Results:**

Ninety-one percent of parents completed the study, 64 in intervention group and 58 in wait-list group. At the sixth month post-training, BMI z-scores were significantly reduced in the intervention group compared to the wait-list group, for the all children (overweight and obese children) and within the obese subgroup ((F(6, 517) = 2.817, *p* = 0.010) and (F(6, 297) = 6.072, *p* < 0.001) respectively. For waist circumference percentile and body fat percentage, the intervention group experienced a significant reduction compared to the wait-list group, within the obese subgroup ((F(6, 297) = 3.998, *p* = 0.001) and within the overweight subgroup (F(6, 201) = 2.526, *p* = 0.022).

**Conclusions:**

The four-month REDUCE intervention programme was effective in reducing childhood adiposity. Further research using this approach needs to be conducted including cost-effectiveness studies before implementing it in a child obesity prevention programme.

**Trial registration:**

Australian New Zealand Clinical Trials Registry: ACTRN12617000844347 (7 June 2017 retrospectively registered). National Medical Research Register, Ministry of Health Malaysia: NMRR-14-685-21,874 (July 2014).

**Electronic supplementary material:**

The online version of this article (10.1186/s12937-018-0379-1) contains supplementary material, which is available to authorized users.

## Background

The prevalence of overweight and obesity among children 5 to 19 years of age was reported to have experienced a tenfold increase worldwide from 11 million in 1975 to 124 million in 2016 [1]. Developing countries are now experiencing such a trend where the relative percentage increase was 59% between the year 1980 and 2013 compared to 41% increase in developed countries [2]. In Malaysia - a developing country - the childhood obesity prevalence increased from 3.9% in 2011 to 11.9% in 2015 [3, 4]. Because childhood obesity is associated with negative health effects, it warrants serious public health attention to prevent further increase in its prevalence [5].

Some ethnic groups are more affected by obesity and in Malaysia, the Chinese have the highest prevalence of obesity (13.0%), followed by Indians (12.6%) and Malays (11.8%). However, in terms of absolute numbers, Malays form the largest group as they are the ethnic majority in Malaysia and thus are more greatly affected in terms of disease burden. The ethnic composition in Malaysia consists of Malay (52.9%), Chinese (27.8%), Indian (13.3%) and other ethnic groups (6%) [6]. Healthy lifestyle programmes that are socio-culturally customized may be more effective [7] and thus this intervention programme was tailored to the Malay ethnicity.

Parents can influence the eating behaviour and physical activities of their children; thus intervention programmes that targeted parents as the agent of change had their roots decades ago [8–19]. However, parents often had other commitments that hindered their adherence to face-face intervention programmes [20]. New approaches to educate parents in nutrition and physical activity have emerged - there is an increasing trend in using social media as a mode of communication among young people and adults. As of April 2018, the most common social networking site worldwide was Facebook (2.2 billion), followed by WhatsApp (1.5 billion), You Tube (1.5 billion) and Facebook Messenger (1.3 billion) [21]. About 76.9% of Malaysians use the Internet, and 97.3% of them are Facebook users [22]. Systematic reviews of experimental studies on health behaviour intervention concluded that this platform could be effectively used to promote behaviour change [23, 24]. Thus, the use of social networking as a platform to provide health education and impart relevant skills could be feasible and appropriate. Studies have incorporated social media in body weight management [25–28] but using this modality among parents to influence their children is a fairly new concept [23, 29]. Currently overweight and obese children in Malaysia are referred for treatment only if they developed complications. Thus developing a child obesity prevention programme which is parent-friendly and provider-friendly is justified and timely to help curb the increasing prevalence of childhood obesity in Malaysia.

The aim of this study is to evaluate the effectiveness of using social media and face-to-face sessions in a family-based intervention on the primary outcome of body mass index (BMI) z-score and secondary outcomes of waist circumference percentile and percentage total body fat. We also assessed parental factors which include parental knowledge, healthy lifestyles, parental feeding style and parental self-efficacy as well as child factors which include children’s eating behaviours, food and beverages consumption (fruit and vegetable, snacks and sugar-sweetened beverages), physical activity and screen time. However, parental factors and child factors are not reported here. We hypothesize that in comparison with the wait-list group, children in the intervention group will have reduced BMI z-score, waist circumference percentile and percentage of total body fat.

## Methods

A detailed elaboration of the methodology of this field trial, has been published elsewhere [30]. However, a summary of the methodology is described here.

### Participants and design

This is a two-armed, parallel, randomized controlled field trial conducted in an urban area in one of the states in Malaysia. An urban area was chosen as the Malaysia National Health and Morbidity Survey (2015) [4] showed that prevalence of childhood obesity was slightly higher in the urban areas than in the rural areas.

All five primary government schools in this area were selected for this study. Before the study was conducted, BMI z-scores of school children in those schools were not available as there was no standardized reporting and referral upon detecting childhood overweight and obesity unless there were complications. Thus the number of children with overweight and obesity were unknown prior to the study. Brochures were sent to all the five schools informing parents about the intervention and the study and requesting them for consent to measure weight and height of their school-going children aged 7 to 10 years from August to September 2014. Those children whose parents gave written consent were screened for BMI z-score eligibility in October 2014. Parents who agreed to participate also provided consent on behalf of their children. Parents whose children with BMI z-score of more than 1 standard deviation (SD) were then invited to participate in the study. Parent-child dyads of Malay ethnicity who were computer literate, had access to the internet, were willing to use social media for interaction and children 7 to 10 years of age were recruited. Parents who reported children having co-morbidities, chronic diseases, physical disabilities, learning disabilities, on medication for chronic illness or participated in other research were excluded.

Individual randomisation was used over cluster design as contamination was expected to be low, because parents in the study setting hardly intermingle. In addition, the distribution of schools across intervention and control groups would be unequal because there are five schools and differences in distribution of sociodemographic characteristics and children with weight problems were unknown prior to the study. Each parent-child dyad from all five schools was number coded by the first author (NA) and sent to a research assistant who performed a computer generated randomization list which allocated parents into intervention or wait-list control groups using a simple randomization procedure of an online software (Research Randomiser) [31] with 1:1 allocation. This ensured the concealment of allocation from the rest of the researchers and participants. The list was then provided to the first author to invite the intervention group for the REDUCE intervention program.

Participating parents were informed that the intervention would be done in stages. So, parents would have the understanding that some of them would be participating earlier than others. Eventually all in the wait-listed control group would undergo the same intervention as parents in the intervention group after the final data collection. Parents in the wait-list control group were not informed of the starting of the intervention for the intervention group. The intervention group was informed not to share their social media experience with other parents; in addition they did not know who were in the wait-listed group, hence minimizing contamination. The face-to-face sessions for the intervention group were conducted in the Faculty of Medicine and Health Sciences, UPM without the knowledge of the wait-listed control group.

The researcher (NA) as the implementer of the intervention was not involved in the measurement of the children’s weight, height, waist circumference and body fat percentage and data entry.

The sample size was based on a power of 80% and a level of significance (α) of 0.05 to detect a BMI z-score difference between intervention and wait-list control groups of 0.24 with standard deviation of 0.48 based on a previous study [32]. The dropout rate after randomization was assumed to be 15% requiring a minimum sample size of 56 parents per arm. The parent-child dyad was based on a 1:1 ratio – one parent to one child. All except two parents had one eligible child who was overweight or obese. For those two parents, the parents were requested to volunteer one other child for the study.

### Intervention

The REDUCE (REorganise Diet, Unnecessary sCreen time and Exercise) intervention programme to impart information and skills was a newly developed programme by the researchers (NA, ZMS and FM) using social cognitive theory (SCT) [33]. The programme trained the parents on children’s nutrition, physical activity, behaviour modification techniques and parenting skills to improve their children’s health behaviours. The SCT posits behaviour as a reciprocal interaction between the person and environmental factors. The person is the child and the environment is the home environment and the parental factors that are conducive to the children’s behaviour change. The child’s ultimate daily targeted behaviours included no consumption of sugar-sweetened beverages (SSB) and unhealthy snacks, intake at least five servings of fruit and vegetables (two servings of fruit and three servings of vegetables), a minimum of 30 min of moderate to vigorous physical activity and a maximum of 120 min of screen time (watching television and playing video games). However, parents and children were empowered to choose which of the five targets to start working towards, first starting with the more manageable to achieve targets and to make small changes, one at a time. The elements of behaviour modification skills in the SCT include self-monitoring, goal setting, self-efficacy, problem solving, relapse prevention, and stimulus control. Parents were encouraged to acquire authoritative parenting skills, practise healthy behaviours and improve self-efficacy of child’s healthy behaviours. The four-month REDUCE intervention programme consisted of 4 weeks of weekly training and 3 months of weekly booster.

The four-week training phase of the REDUCE intervention module was comprised of 8 units; 2 units were delivered through half-day face-to-face sessions (session one) followed by 2 units delivered weekly via Facebook for 2 weeks, and finally the last 2 units delivered via half-day face-to-face sessions (session two). The face-to-face sessions were conducted at the Faculty of Medicine and Health Sciences, University Putra Malaysia. All 4 units delivered in the face-to-face sessions were subsequently uploaded on the Facebook after each session. The training contents are summarized in Table 1. All the training sessions were delivered by the first author (NA) who is a public health physician except for the exercise tips in unit 8 which was conducted by a sports medicine specialist. All units were delivered to parents only, except for units 7 and 8 which were delivered to parents and children.

**Table 1:** Contents of REDUCE intervention module

Week	Unit	Approach	Themes	Behaviour modification techniques
1	1	Face-to-face session one	Introduction, obesity overview, parenting skills and role modelling	Goal setting, self-monitoring, self-efficacy, problem solving and stimulus control
	2		Sugar-sweetened beverages	
2	3	Facebook	Fruits and vegetables	Goal setting, self-monitoring, self-efficacy, problem solving and stimulus control
	4		Unhealthy snacks	
3	5		Physical activity	
	6		Screen time	
4	7	Face-to-face session two	Risky situations and review of performance	Relapse prevention
	8		Further roles and actions, exercise tips and success stories	

The booster phase of the REDUCE intervention programme were weekly one-hour sessions using parents’ dedicated WhatsApp group that lasted for 12 weeks. The aims of the booster phase were to strengthen parents’ knowledge and skills in promoting the targeted behaviours of the REDUCE programme. In this phase the first author (NA) posted on WhatsApp key information and skills provided in the training phase but in the form of a poster, responded to any queries by parents and provided feedback on the adiposity progress of the children based on the measurements taken. Parents were encouraged to enquire and discuss with the researcher (NA) and to interact with other parents in the intervention group in order to promote programme adherence and maintain motivation using this platform. The total contact time between researcher and participants was 22 h. Programme adherence was assessed by percentage of parents who attended the face-to-face sessions, and accessed the information in the Facebook as well as messages in the WhatsApp. Parents’ responses were also compared between Facebook and WhatsApp.

The wait-list control group received the intervention after the completion of the final 6-month follow-up. To minimize attrition among intervention and wait-list control groups, small incentives (mainly stationeries) were given to the children each time they participated in the physical measurements and returned the parent-administered questionnaires.

### Parents’ involvement

In the pilot testing of the REDUCE intervention module, Malay parents of obese and overweight primary school children were involved in providing feedback on the module’s duration, mode of delivery and the performance feedback of the children’s progress over an eight-week training programme delivered only via Facebook.

In the randomized controlled trial, parents in the intervention group were informed of their children’s progress via WhatsApp after each data collection immediately post-training, at 3 months and 6 months post-training while parents in the wait-listed group received feedback at similar times after the study ended. Feedback was obtained from parents in the intervention group regarding the burden or otherwise of the intervention.

### Measures

The primary outcome was BMI z-score while waist circumference percentile and percentage of total body fat were secondary outcomes. BMI z-score which was determined using the WHO Anthroplus software [34] with weight in kg, height in cm, gender and age. Height was measured using a portable Seca stadiometer to the nearest 0.1 cm with bare feet. Weight was measured using the Omron Karada Scan (model HBF 212) to the nearest 0.1 kg with light clothing on and empty pockets.

Waist circumference was measured using Seca non-extensible tape meter at the approximate midpoint between the lower margin of the last palpable rib and the top of the iliac crest at the end of normal expiration [35]. The waist circumference was then converted to waist circumference percentile for Malaysian children by reading off the table on percentile values of WC (cm) by age and gender [36]. Percentage of body fat was measured after weight measurement using the same scale to the nearest 0.1%. All physical measurements were performed by trained research assistants who were blinded regarding the allocation status of these children. The implementer of the intervention (NA) was not involved in any of these measurements. Each type of measurement was assigned to a designated trained research assistant to minimize inter-rater measurement bias. For example, a research assistant who did waist circumference only measured waist circumference. Assessments were completed at baseline, immediate post-training, 3 months and 6 months post-training.

Sociodemographic information was collected at baseline comprising of child’s data on birth date, gender, number of siblings, order in the family and parents’ data on marital status, education level and income. Parents’ self-report on weight and height were also collected at baseline to determine parents’ BMI.

### Statistical analysis

Data were analysed using SPSS version 22.0. Tests for normality were done using skewness, kurtosis, and graphs (histogram with normal distribution curve, whisker boxplot, and Q-Q and P-P plots) before further analyses were conducted. Non-normally distributed data were log transformed first before further analysis. Differences between intervention and wait-list control at baseline were tested using independent t-test (continuous variables) or chi-square test or Fischer’s exact test (categorical variables) to examine the homogeneity between groups. All follow-up outcomes were analysed with intention-to-treat analysis.

Changes in BMI z-score, waist circumference percentile and percentage of total body fat were compared between intervention and wait-list control groups using independent t-test for continuous variables. The effectiveness of the intervention was evaluated using generalized linear mixed modelling adjusted for baseline covariates. Covariates included were variables that could affect body weight - child’s age, child’s gender, parents’ BMI, parents’ education, family income, and child’s adiposity baseline measurements (BMI z-score, waist circumference percentile and percentage body fat). Subgroup analysis was conducted for overweight and obese children. The level of significance was set at α = 0.05, and the null hypothesis rejected when *p* ≤ 0.05. The CONSORT checklist of this study is in the Additional file 1.

## Results

**Fig. 1:**
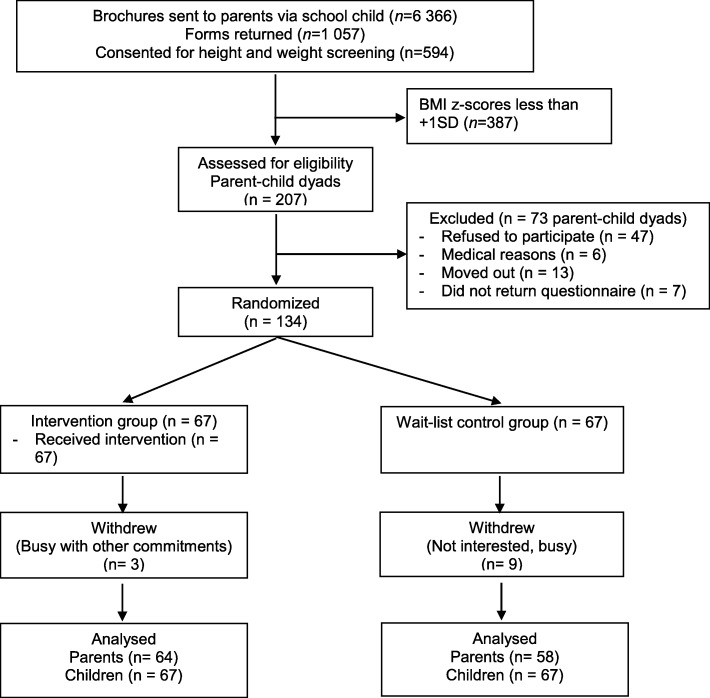
CONSORT flow diagram of parent-child’s participation throughout study period

One hundred and twenty-two parents completed this study giving an overall response rate of 91% among parents (64 parents in the intervention group and 58 parents in the wait-list control group were available for analysis). Parents who did not return the questionnaire after defaulting three follow-up sessions were considered dropouts. There was no significant difference between parents who remained in the study and parents who dropped out in terms of sociodemographic characteristics, parental BMI and the anthropometric measurements of their children. There was complete data for anthropometry and body fat percentage of all the children at all the four time points. The CONSORT flow diagram of parent-child participation is shown in Fig. 1.

Table 2 shows the baseline measurements of the respondents. There was no significant difference between the intervention and control groups at baseline. There was no unintended effect reported by the participants in the intervention and wait-list groups.

**Table 2:** Baseline characteristics of the sample

Characteristics	Intervention (n = 67)		Wait-list control (*n* = 67)		*P* value
	n (%)	Mean ± SD	n (%)	Mean ± SD	
Parent					
Age (years)		39.8 (3.6)		41.3 (5.7)	0.079
Gender - female	39 (58.2)		37 (55.2)		0.862
BMI (kg/m^2^)		27.4 (4.41)		27.8 (4.27)	0.622
Child					
Age (years)		9.6 (1.2)		9.6 (1.2)	0.826
Gender - female	40 (59.7)		38 (56.7)		0.861
Height (cm)		136.1 (8.6)		135.6 (9.2)	0.753
Weight (kg)		47.0 (10.5)		48.2 (12.0)	0.537
BMI		25.2 (3.5)		25.7 (3.9)	0.272
BMI z-score		2.0 (0.4)		2.1 (0.4)	0.381
BMI z-score category					0.861
Overweight	28 (41.8)		27 (40.3)		
Obese	39 (58.2)		40 (59.7)		
Waist circumference (cm)		77.7 (8.8)		78.8 (9.1)	0.465
Waist circumference percentile		90.2 (8.0)		91.3 (7.0)	0.410
Total body fat		37.9 (4.2)		37.6 (4.1)	0.740

### Programme adherence

96.9% of parents participated in WhatsApp and 81.3% in Facebook respectively, compared to 68.8% for session one and 42.2% for session two of the face-to-face sessions. Since all the material used in the face-to-face sessions was uploaded on the Facebook after each session, the contents of the face-to-face sessions thus were available for all parents in the intervention group. Parents’ adherence in WhatsApp was by counting the number of times they accessed the messages. Table 3 shows parents’ participation and characteristics in WhatsApp and Facebook. One quarter of the parents’ responses (82/356) expressed support for the programme. Examples of texted support included: expressing thanks for having the programme, welcoming other group members, texting phrases such as ‘to make healthy and happy family’ and congratulating group members who managed to reduce their children’s BMI. One fifth of the responses (77/356) shared the parents’ or their child’s progress. For example, they reported on their children’s improvement in reducing sugar-sweetened beverages, their ways of doing so and their children’s activities. Another one fifth of the responses (75/356) gave simple replies. Examples included thank you, thumbs up and a smiley emoji.

**Table 3:** Parents’ participation and characteristics in WhatsApp and Facebook

Characteristics	WhatsApp n (%)	Facebook n (%)
Initial number of parents at start of study, n (%)	64 (96)	64 (96)
Number of parents who left group, n (%)	3 (4)	3 (4)
Total responses by parents	356	135
Total posts and responses by researcher	179	34
Responses by parents:		
Request clarification on the post	9	–
Support programme	82	45
Sharing of their problems or progress or child’s progress	77	8
Request regarding child’s progress	20	3
Sharing information about food and physical activity	24	2
Simple replies (e.g. thank you, thumbs up, smiley emoji)	75	73
Suggestions to improve children’s obesity issue at school level and national level	6	–
Enquiry about other health issues	4	–
Others	59	4
Responses per parent:		
1–5	35	36
6–10	18	14
≥11	11	14

### Impact on children’s adiposity

Changes in children’s adiposity for the intervention and wait-list control groups over the course of the study are shown in Table 4. There were increases in mean differences between intervention and wait-list control groups observed in BMI z-score and waist circumference percentile over the study period and these differences were significant at the end of 6 months (*p* = 0.045 and *p* = 0.021 respectively).

**Table 4:** Changes in child’s adiposity between intervention and wait-list groups from baseline to 6-month post-training

Variables	Data (*N* = 134) Means ± SD			
	Baseline	Immediately post-training	3-month post-training	6-month post-training
BMI z-scores				
Intervention group (*n* = 67)	2.05 ± 0.40	2.03 ± 0.38	1.99 ± 0.41	1.95 ± 0.45
Wait-list group (*n* = 67)	2.11 ± 0.39	2.10 ± 0.36	2.11 ± 0.35	2.09 ± 0.35
t-test (*p*-value)	- 0.879 (*p* = 0.381)	−1.158 (*p* = 0.249)	−1.730 (*p* = 0.086)	−2.025 (p = 0.045)^a^
Mean difference	− 0.06	− 0.07	−0.11	− 0.14
95% CI for mean difference	−0.194 to 0.075	− 0.200 to 0.052	−0.244 to 0.016	- 0.278 to - 0.003
Waist circumference percentile				
Intervention group (*n* = 67)	90.21 ± 7.98	91.09 ± 7.71	90.06 ± 9.56	89.37 ± 9.26
Wait-list group (*n* = 67)	91.28 ± 7.04	93.82 ± 4.31	92.25 ± 6.11	92.55 ± 6.20
t-test (*p*-value)	− 0.826 (*p* = 0.410)	− 2.532 (*p* = 0.013)^a^	− 1.583 (*p* = 0.116)	−2.335 (p = 0.021)^a^
Mean difference	− 1.07	−2.73	− 2.19	−3.18
95% CI for mean difference	−3.647 to 1.498	−4.871 to −0.592	−4.940 to 0.552	−5.876 to − 0.482
Percentage of body fat				
Intervention group (*n* = 67)	37.87 ± 4.20	37.68 ± 4.14	36.66 ± 4.75	36.75 ± 4.75
Wait-list group (*n* = 67)	37.63 ± 4.09	37.23 ± 4.50	37.65 ± 4.25	37.25 ± 4.46
t-test (p-value)	0.333 (*p* = 0.740)	0.603 (*p* = 0.548)	−1.263 (*p* = 0.209)	− 0.630 (*p* = 0.530)
Mean difference	0.24	0.45	−0.98	−0.50
95% CI for mean difference	−1.178 to 1.654	− 1.027 to 1.927	−2.524 to 0.557	− 2.077 to − 1.074

^a^Significant at *p* ≤ 0.05, *SD* standard deviation, *CI* confidence interval

Table 5 shows the changes in adiposity between baseline and six-month follow-up for the whole sample, overweight and obese subgroups. The mean decrease in BMI z-score was 0.10 ± 0.26 for whole sample and 0.11 ± 0.14 for obese subgroup in the intervention group. The wait-list control group had higher increment in mean waist circumference percentile at six-month follow-up (2.9 cm ± 8.5) compared to intervention group (1.5 cm ± 7.2). There was a higher reduction in within-group change for percentage of total body fat at six-month follow-up for overweight children in the intervention group (2.5% ± 6.7) compared to the wait-list group. However, this change was not significant (*p* = 0.065).

**Table 5:** Comparison of changes in adiposity within groups between baseline and six-month follow-up

	Mean difference ± SD baseline vs 6-month^c^	t	*p*-value ^a^
BMI z-score			
Whole sample			
Intervention group (*n* = 67)	0.100 ± 0.257	3.190	0.002^b^
Wait-list group (*n* = 67)	0.019 ± 0.172	0.907	0.368
Overweight children			
Intervention group (*n* = 28)	0.087 ± 0.367	1.257	0.220
Wait-list group (*n* = 27)	− 0.067 ± 0.185	−1.887	0.070
Obese children			
Intervention group (*n* = 39)	0.109 ± 0.136	5.022	< 0.001^b^
Wait-list group (*n* = 40)	0.077 ± 0.138	3.545	0.001^b^
Waist circumference percentile			
Whole sample			
Intervention group (*n* = 67)	1.373 ± 11.079	1.014	0.314
Wait-list group (*n* = 67)	−1.343 ± 9.019	−1.219	0.227
Overweight children			
Intervention group (*n* = 28)	5.357 ± 14.095	2.011	0.054
Wait-list group (*n* = 27)	0.963 ± 9.382	0.533	0.598
Obese children			
Intervention group (*n* = 39)	−1.487 ± 7.207	−1.289	0.205
Wait-list group (*n* = 40)	− 2.900 ± 8.533	−2.149	0.038^b^
Percentage of total body fat			
Whole sample			
Intervention group (*n* = 67)	0.912 ± 6.053	1.234	0.222
Wait-list group (*n* = 67)	0.655 ± 6.694	0.801	0.426
Overweight children			
Intervention group (*n* = 28)	2.451 ± 6.736	1.926	0.065
Wait-list group (*n* = 27)	1.963 ± 5.961	1.711	0.099
Obese children			
Intervention group (*n* = 39)	−0.193 ± 5.330	−0.226	0.822
Wait-list group (*n* = 40)	−0.227 ± 7.083	−0.203	0.840

^a^Using paired t-test; SD: standard deviation

^b^Significant at *p* ≤ 0.05;

^c^This difference is baseline minus 6 month, with minus value depicting a higher measurement at 6-month post-training compared to baseline

**Fig. 2:**
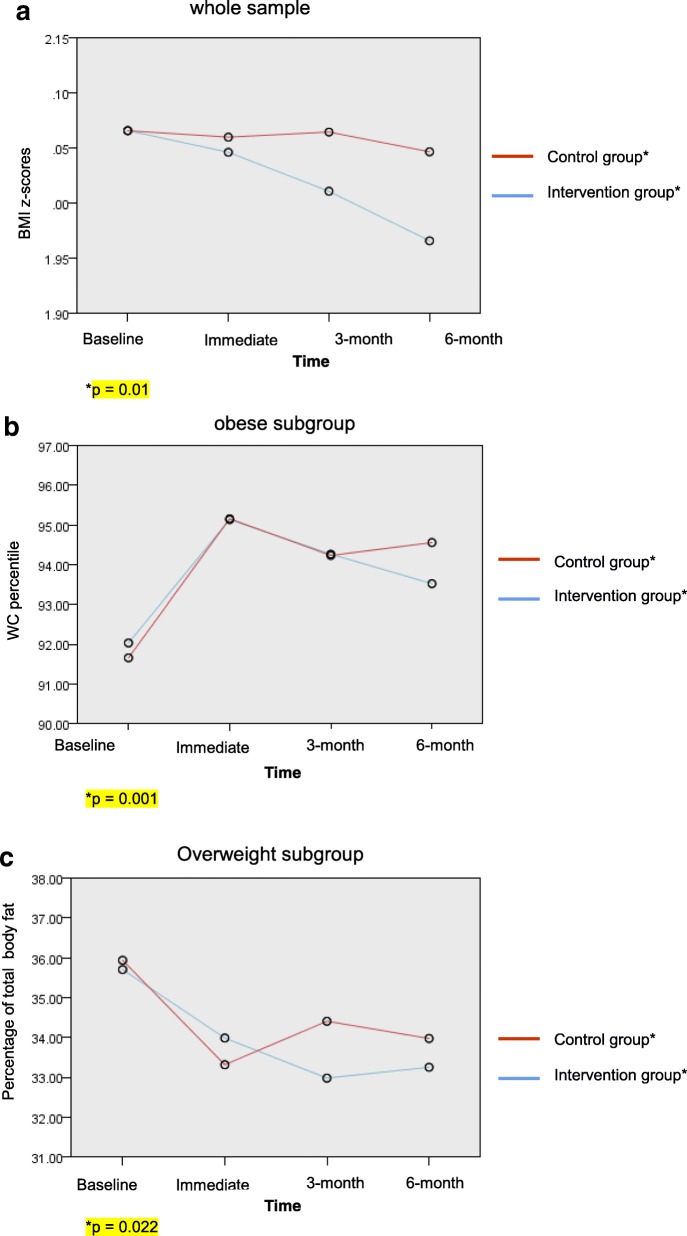
Changes in BMI z-scores, waist circumference percentile and body fat percentage from the mixed model analysis, between baseline and at 6 months

Results from the mixed model analysis (Table 6) showed that at the end of 6 months, significantly higher proportion of children in the intervention group than in the wait-list group had reduced BMI z-score for all (F(6, 517) = 2.817, *p* = 0.010). Among obese children (F(6, 297) = 6.072, p = < 0.001), more children in the intervention group had reduced their waist circumference percentile (F (6, 297) = 3.998, *p* = 0.001) compared to the control group, while more overweight children in the intervention group had reduced their body fat percentage compared to the control group (F (6, 201) = 2.526, *p* = 0.022). Figure 2 shows changes in BMI z-scores, waist circumference percentile and body fat percentage from the mixed model analysis, between baseline and at 6 months (p = 0.01, p = 0.001 and p = 0.022 respectively).

**Table 6:** Comparison of anthropometric measurements and body fat percentage between study groups, controlling for covariates

Variable	Parameter	F	df1	df2	p-value^a^
BMI z-scores					
Whole sample	Group	7.050	1	517	0.008^b^
(*n* = 134)	Group x Time	2.817	6	517	0.010^b^
Overweight subgroup	Group	9.341	1	201	0.003^b^
(*n* = 55)	Group x Time	1.218	6	201	0.299
Obese subgroup	Group	0.033	1	297	0.855
(*n* = 79)	Group x Time	6.072	6	297	< 0.001^b^
Waist circumference percentile					
Whole sample	Group	13.935	1	517	< 0.001^b^
(*n* = 134)	Group x Time	1.410	6	517	0.209
Overweight subgroup	Group	30.245	1	201	< 0.001^b^
(*n* = 55)	Group x Time	1.683	6	201	0.127
Obese subgroup	Group	0.084	1	297	0.772
(*n* = 79)	Group x Time	3.998	6	297	0.001^b^
Body fat percentage					
Whole sample-	Group	1.454	1	517	0.228
(*n* = 134)	Group x Time	0.802	6	517	0.569
Overweight subgroup	Group	0.575	1	201	0.449
(*n* = 55)	Group x Time	2.526	6	201	0.022^b^
Obese subgroup	Group	0.093	1	297	0.760
(*n* = 79)	Group x Time	0.628	6	297	0.708

^a^Using generalized linear mixed model adjusted for child’s age, child’s gender, parents’ body mass index, mother’s education, father’s education, family income, and child’s baseline data (BMI z-score, waist circumference percentile and body fat percentage respectively)

^b^Significant at *p* ≤ 0.05

We received positive feedback from parents regarding the use of social media. Parents felt social media was convenient to use and facilitated direct communication with the researcher who provided the needed support. It also made possible discussion to solve problems that parents may face regarding the study, and enabled performance feedback to the parents.

## Discussion

In this randomized controlled field trial, our results suggest that the use of social media and face-to-face sessions in the family-based, theory-driven intervention for management of childhood obesity can decrease children’s adiposity (BMI z-score, waist circumference percentile and percentage of body fat). No adverse events or unintended adverse consequences of the intervention were reported by the participants.

These results showed almost similar magnitude of decrease in BMI z-score found in Cochrane meta-analysis among 37 studies for children aged 6–12 years with mean effect of − 0.15 (95% CI: -0.23 to − 0.08) [37]. This difference is expected to have clinical benefits as a study by Kolsgaard et al. (2011) showed that even a modest reduction in BMI z-score (≥0.00 - < 0.1) was associated with significant improvement in several cardiovascular risk factors i.e. improved insulin and insulin resistance, total cholesterol, LDL cholesterol and total/HDL cholesterol ratio [38].

A recent systematic review and meta-analysis of eight studies examining the effectiveness of family-based eHealth intervention among overweight and obese children and adolescents found that the difference in BMI or BMI z-score between the intervention and control groups at post-intervention were not statistically significant [39]. The REDUCE intervention also produced a greater reduction in BMI z-scores than some of the face-to-face only interventions for parents of overweight and obese children [40–44] that measured similar outcomes. We postulate that a combination of social media and face-to-face intervention may have produced more favourable results compared with standalone intervention of either internet/social media or face-to-face intervention. The results of the REDUCE intervention compare favourably with a more intensive intervention [32] that involved 18 two-hour face-to-face sessions delivered over 9 weeks. There are, however, some face-to-face interventions that have produced larger group differences than the REDUCE intervention [12, 45]. Their interventions however, involved more sessions than the REDUCE intervention.

Subgroup analysis showed that in the intervention group, obese children had significantly lesser increment at the end of the 6-month post-training in waist circumference percentile whereas overweight children had significantly reduced percentage of total body fat compared to the wait-list group. The result of our study was comparable to those of a face-to-face intervention study by Sacher et al. [32]. Their intervention resulted in a − 4.1 cm significant difference in waist circumference between intervention (77.7 ± 7.2 cm) and control groups (82.0 ± 8.6 cm) over a six-month period. Our study showed a − 4.1 cm difference in waist circumference between intervention (71.49 ± 6.04 cm) and wait-list (75.63 ± 6.49 cm) groups for overweight children at six-month follow-up even though it was not significant, most likely due to insufficient power. The study by Reinehr et al. (2010) among overweight children also found a significant difference between the waist circumference of the intervention and control groups [46]. However, other conventional family-based behavioural treatment of childhood obesity found no difference in waist circumference between the intervention and control groups [41, 43].

The REDUCE intervention had a greater impact on percentage body fat than another Internet-based family intervention [47] and a conventional family intervention [32] both of which found no difference between intervention and wait-list groups after the same period of follow-up. However, they did not perform subgroup analysis based on obesity status (overweight or obese). The children in our study had high values of percentage body fat with a mean value of more than 35%. Yamashita et al. (2012) had suggested the cut-off point for cardiovascular risk for Asian adults to be 20.3% [48]. No cut-off point as yet has been developed for children but the values found in the current study are much higher compared with the adult values, putting these children potentially at elevated cardiovascular risk later in life.

Our study showed a high response rate in the intervention group, with higher rates of parents’ participation in the WhatsApp and Facebook sessions compared to face-to-face sessions, indicating that the social media approach is better received than the face-to-face sessions. This is possibly due to the rigid nature of the face-to-face sessions where parents had to attend the sessions within the allocated time, making these sessions least attended. However, omitting the face-to-face approach could possibly have affected the results of this study. In comparing parents’ participation in the two types of social media i.e. Facebook and WhatsApp, results showed a greater participation in WhatsApp. We postulate that this was due to the longer duration of contact with parents (3 months) in WhatsApp compared to Facebook (1 month). Facebook has been reported to be the most commonly used social medium in delivering health interventions compared to other social media [23]. However, the use of WhatsApp in health settings has not been widely established as yet. A study by Cheung et al. evaluated the effectiveness of using WhatsApp or Facebook to prevent smoking relapse in recent quitters and found that respondents were more likely to participate using WhatsApp (76%) than Facebook (42%) [49]. WhatsApp may be gaining greater acceptance than Facebook because its features are more convenient and easier to use without requiring logging in, or a username and password [49–51]. Apart from notifying smartphone users about incoming messages, WhatsApp allows them to make quick and simple responses and the messages were encrypted [50, 51]. During the training phase of this intervention, Facebook was used because at that time, WhatsApp did not support the uploading of documents. Even though this function was introduced when the booster phase began, nevertheless, downloading inordinately large documents via WhatsApp caused mobile phones to cease to respond.

The intervention incorporated setting realistic and achievable goals for the children. However, there are several limitations to this study. Generalizability is limited due to the sample being among urban, Malay and educated parents. The respondents could possibly have been highly motivated parents. Parents’ BMI was self-reported with the possibility of recall bias being introduced. The poor attendance in face-to-face sessions could have affected the effectiveness of the intervention. To minimize this effect, the respective materials of the module were uploaded to Facebook, and the parents’ adherence in Facebook was assessed by the number and percentage who accessed this module. However, we were not able to determine the association between adherence and outcomes as we did not measure individual adherence scores. Another limitation is that the effect of other influences outside the home environment was not controlled for. Lastly, this study was retrospectively registered. However, there was no element of selective reporting for this study, with the original protocol uploaded in its entirety. Future research should be conducted to have a longer follow-up with a cost-effectiveness study before implementing this programme in child obesity prevention programme.

## Conclusions

The REDUCE intervention programme shows that it is possible, in an urban Malay community, to reduce childhood adiposity in primary school children by using a combined face-to-face approach and social media in educating parents on child nutrition, physical activity, behaviour modification techniques and parenting skills. This study thus provides evidence of the potential usefulness of using face-to-face interaction and social media for a family-based intervention in urban community settings to help curb the epidemic of childhood obesity. However, a longer duration of study is needed to ascertain the sustainability of the intervention. A cost-effectiveness study is recommended before incorporating this intervention in a child obesity prevention programme.

## Additional files

Additional file 1: 2017 CONSORT checklist of information to include when reporting a randomized trial assessing nonpharmacologic treatments (NPTs). (DOCX 51 kb)

## Abbreviations

BM: Body mass index
REDUCE: Reorganise diet, unnecessary screen time and exercise
SCT: Social cognitive theory
